# Calcium Signaling Mediates Cell Death and Crosstalk with Autophagy in Kidney Disease

**DOI:** 10.3390/cells10113204

**Published:** 2021-11-17

**Authors:** Bo Ning, Chuanzhi Guo, Anqi Kong, Kongdong Li, Yimin Xie, Haifeng Shi, Jie Gu

**Affiliations:** 1 School of Life Sciences, Jiangsu University, Zhenjiang 212013, China; 2211917021@stmail.ujs.edu.cn (B.N.); 2212117016@stmail.ujs.edu.cn (C.G.); 2211917011@stmail.ujs.edu.cn (A.K.); 2212017010@stmail.ujs.edu.cn (K.L.); shihf@ujs.edu.cn (H.S.); 2 Affiliated Hospital of Jiangsu University—Yixing Hospital, Yixing 214200, China; staff1999@yxph.com; 3 School of Food and Biological Engineering, Jiangsu University, Zhenjiang 212013, China

**Keywords:** Ca^2+^ signaling, cell death, autophagy, kidney diseases

## Abstract

The kidney is an important organ for the maintenance of Ca^2+^ homeostasis in the body. However, disruption of Ca^2+^ homeostasis will cause a series of kidney diseases, such as acute kidney injury (AKI), chronic kidney disease (CKD), renal ischemia/reperfusion (I/R) injury, autosomal dominant polycystic kidney disease (ADPKD), podocytopathy, and diabetic nephropathy. During the progression of kidney disease, Ca^2+^ signaling plays key roles in various cell activities such as necrosis, apoptosis, eryptosis and autophagy. Importantly, there are complex Ca^2+^ flux networks between the endoplasmic reticulum (ER), mitochondria and lysosomes which regulate intracellular Ca^2+^ signaling in renal cells and contribute to kidney disease. In addition, Ca^2+^ signaling also links the crosstalk between various cell deaths and autophagy under the stress of heavy metals or high glucose. In this regard, we present a review of Ca^2+^ signaling in cell death and crosstalk with autophagy and its potential as a therapeutic target for the development of new and efficient drugs against kidney diseases.

## 1. Introduction

Through gradual evolutionary development, Calcium has become one of the most important metal elements in living organisms. With a wide variety of biological functions in living creatures, calcium ions (Ca^2+^) are involved in almost every process from birth to death [1].

Ca^2+^ is primarily stored in bones in the form of CaPO_3_ (hydroxyapatite), where it plays a structural role and also can be dissolved to serve as a source of Ca^2+^ in the blood [2]. In addition, Ca^2+^ is a ubiquitous, multifunctional signaling molecule that controls a wide variety of life processes including muscle contraction, neuronal delivery, hormone secretion, organelle communication, cell movement, fertilization, and cell growth. Because of the critical role of Ca^2+^ in these vital activities, cellular Ca^2+^ concentration is strictly regulated, and dysfunction of cellular Ca^2+^ homeostasis is closely associated with a great number of diseases such as heart and hepatic diseases, aging, and type 2 diabetes, as well as certain cancers [3,4,5].

The kidney is an indispensable organ for maintaining body Ca^2+^ homeostasis; the Ca^2+^ signaling system in kidney cells regulates cellular processes and decides cell fate, including cell proliferation, apoptosis, necrosis and autophagy, all of which are associated with kidney disease [6,7]. Ca^2+^ homeostasis has a certain influence on renal function and the occurrence and development of a series of nephropathies including acute kidney injury (AKI), chronic kidney disease (CKD), renal ischemia/reperfusion (I/R) injury, autosomal dominant polycystic kidney disease (ADPKD), podocytopathy, and diabetic nephropathy, which will be discussed in the following sections.

Based on the significance of Ca^2+^ signaling in the regulation of cell fate in the context of kidney disease, this review focuses on cell death determined by Ca^2+^ signaling in kidney cells and erythrocytes. We present a general overview of Ca^2+^ signaling in kidney disease, as well as some types of cell death that are regulated by Ca^2+^ signaling. In addition, targets of Ca^2+^ signaling to prevent kidney disease are discussed based on the available data.

## 2. Relationship between Ca^2+^ Signaling and Various Forms of Cell Death in Kidney Cells

Ca^2+^ not only regulates the normal life activities of cells and organisms, but has also been found to play an important role in the regulation of various cell deaths. Early death of necrotic cells is associated with intracellular Ca^2+^ overload. It has also been found that apoptosis is controlled by Ca^2+^ signaling, and other cell activity, including cell death of erythrocytes (eryptosis), has also been shown to be regulated by Ca^2+^. Moreover, Ca^2+^ signaling is an important mediator of autophagy, which plays dual roles in cell survival and death. The numerous Ca^2+^ channels on cell membranes, endoplasmic reticulum (ER), and mitochondria, as well as alterations in lysosomes, modulate cytosolic Ca^2+^ levels associated with cell necrosis, apoptosis, eryptosis and autophagy. We searched for original research articles from the past two decades using the keywords “Ca^2+^” and “kidney disease” or “kidney injury”, and “necrosis”, or “apoptosis”, or “eryptosis”, or “autophagy”, and/or “endoplasmic reticulum (ER)”, and/or “mitochondrial”, and/or “lysosome” in the PubMed database, then sorted by “best match” with the “summary” format. For review articles, we referenced the information as mentioned in the study by Aromataris et al., such as by number of databases sourced and searched, number of studies, type and country of origin of the studies included in each review, and method of synthesis/analysis employed to synthesize the evidence [8].

### 2.1. Ca^2+^ Mediates Necrosis in Kidney Injury

Necrosis is early cell death associated with intracellular Ca^2+^ overload, and has been found to be regulated by intracellular Ca^2+^ release and/or extracellular Ca^2+^ influx. Methylglyoxal, a physiological glucose metabolite, induces necrotic cell death in Madin-Darby canine kidney (MDCK) renal tubular cells by stimulating both extracellular Ca^2+^ influx and ER Ca^2+^ release [9]. Oxidant tert-butyl hydroperoxide (TBHP) causes ER lipid peroxidation and Ca^2+^ release in isolated rabbit kidney cortex microsomes, and TBHP-induced necrotic cell death is reduced by an ER Ca^2+^ reuptake pump inhibitor, thapsigargin [10]. *Bothrops leucurus* venom increases cytosolic Ca^2+^ in a concentration-dependent manner, participating predominantly in necrotic cell death, which contributes to the acute renal failure and nephrotoxicity in the isolated perfused kidney and cultured renal tubular epithelia [11]. This suggests that intracellular Ca^2+^ release mediates necrotic cell death induced by stress in kidney cells.

Transient receptor potential ankyrin 1 (TRPA1), a redox-sensing Ca^2+^-influx channel, is upregulated in the renal tubules of patients with acute tubular necrosis, which is significantly associated with high incidence of renal function recovery [12]. Internalization of stone-forming calcium crystals such as calcium phosphate (CaP), calcium oxalate (CaOx), and CaP  +  CaOx in renal tubular cells via endocytosis promotes necrosis by store-operated Ca^2+^ entry (SOCE), which causes prolonged Ca^2+^ entry and leads to a continuous rise in intracellular Ca^2+^ levels [13]. This shows that the stimulation of Calcium-Sensing Receptor (CaSR) by melamine causes a sustained rise in intracellular Ca^2+^ levels, leading to enhanced ROS production and a dose-dependent increase in apoptotic and necrotic cell death in the porcine renal proximal tubule cell line, LLC-PK1 [14]. However, the activation of CaSR by L-ornithine can protect from H_2_O_2_-induced necrosis in proximal tubular cells and reduce subsequent acute kidney injury (AKI), which is mediated by transient receptor potential canonical (TRPC)-dependent receptor-operated Ca^2+^ entry [15]. Due to this nephroprotective role of L-ornithine, it can be a useful treatment to reverse kidney diseases. These results suggest that the Ca^2+^-influx channels which mediate Ca^2+^ entry have dual roles in renal epithelial cells. Sustained extracellular Ca^2+^ entry can contribute to renal cell necrosis, while modulation of appropriate extracellular Ca^2+^ entry may protect against kidney injury.

In addition, Na^+^/Ca^2+^ exchange is one of the main factors in reversal of intracellular Ca^2+^ overload in animals with ischemia/reperfusion injury and contrast-induced acute kidney injury [16,17]. KB-R7943, an inhibitor of Na^+^/Ca^2+^ exchange, significantly attenuates renal damage and Ca^2+^ deposition in necrotic tubular epithelium, suggesting that KB-R7943 may be an effective therapeutic agent for acute kidney injury. The use of chemotherapy with ifosfamide is limited by the nephrotoxicity caused by its metabolite chloroacetaldehyde. Chloroacetaldehyde induces sustained elevation of intracellular free Ca^2+^ by inhibiting the Na^+^/Ca^2+^ exchanger, which contributes to necrotic rather than apoptotic cell death and finally leads to nephrotoxicity [18]. Both KB-R7943 and chloroacetaldehyde contribute to the inhibition of Na^+^-dependent extrusion of intracellular Ca^2+^; however, the former did not induce nephrotoxicity, which might due to different Ca^2+^ load levels in the kidney cells.

### 2.2. Ca^2+^ Signaling-Mediated Apoptosis Contributes to Kidney Disease

Apoptosis, or autonomous programmed cell death, is an important process for the maintenance of the stability of the internal environment, and helps an organism better adapt to its living environment. In kidney injury, apoptosis usually occurs in association with necrosis, and Ca^2+^ signaling acts as an important regulator of apoptosis. Ca^2+^ overload leads to necrotic or apoptotic death in renal ischemia–reperfusion (I/R) injury [19]. Sustained ER Ca^2+^ release induces ER stress and oxidative stress and leads to apoptosis in glomerular mesangial cells, which contributes to the progression of CKD [20]. CaSR is a pleiotropic receptor capable of regulating Ca^2+^ homeostasis, and plays important roles in kidney cells and cancers [21]. Activation of adiponectin receptors by AdipoRon and activation of CaSR by cinacalcet increases intracellular Ca^2+^ levels, which inhibits apoptosis in the kidneys induced by high glucose and ameliorates glomerular endothelial cell and podocyte injury in type 2 diabetes-associated diabetic nephropathy [22,23]. This suggests that regulation of cytosolic Ca^2+^ can control the apoptosis of kidney cells.

#### 2.2.1. Endoplasmic Reticulum (ER) Ca^2+^ Signaling Mediates Apoptosis in Kidney Disease

It is well known that the ER is the largest pool of Ca^2+^ in cells, and it plays an important role in regulating intracellular Ca^2+^ balance; however, its dysregulation causes cell apoptosis. The ER Ca^2+^ homeostasis is mainly controlled by two Ca^2+^ releasing channels, inositol 1,4,5-trisphosphate receptor (IP3R) and ryanodine receptor (RyR), and one Ca^2+^ reuptake channel, sarco/endoplasmic reticulum Ca^2+^-ATPase (SERCA). Dysfunctions of these ER Ca^2+^ channels cause a variety of kidney diseases, including ischemia/reperfusion (I/R)-induced tubular injury, autosomal dominant polycystic kidney disease (ADPKD), podocytopathy, and diabetic nephropathy [24]. It is reported that I/R induces renal tubule apoptosis when activation of IP3R starts a cascade of Ca^2+^ release from the ER store [25]. In addition, inhibition of the phosphorylation of IP3R1 at S2681 increases IP3-induced Ca^2+^ release in cystic cells, which contributes to increased apoptosis in ADPKD [26]. Stressed podocytes in the ER undergo phosphorylation of RyR2 at S2808, causing ER Ca^2+^ efflux through leaky RyR2 which in turn activates cytosolic protease calpain 2, leading to podocyte injury and podocyte apoptosis [27]. The evidence implies that significant reductions in SERCA2 activity and expression in the kidney contribute to the development of diabetic nephropathy through ER stress-mediated apoptotic pathways [28]. These results suggest that Ca^2+^ channels in the ER play crucial roles in maintaining intracellular Ca^2+^ homeostasis in kidney cells, and that it is for this reason that renal cell apoptosis and kidney dysfunction contribute to the development of kidney disease.

#### 2.2.2. ER-Mitochondrial Ca^2+^ Signaling Mediates Apoptosis in Kidney Disease

Mitochondria represent another intracellular Ca^2+^ store, and Ca^2+^ signaling mediates the crosstalk between the ER and the mitochondria. Ca^2+^ released by IP3R under ER stress is sequestered by mitochondria for mitochondrial respiration and ATP production, and for regulating cell survival. Mitochondrial release of calpain or caspase-3 promotes the change of ER Ca^2+^ channels, including cleavage IP3R or oxidizing RyR2. These changes may dramatically increase mitochondrial and cytosolic Ca^2+^ levels and augment apoptotic cell death [6]. In addition, cytochrome c released by mitochondria can bind to IP3R, thereby blocking its functional inhibition and increasing the cytoplasmic Ca^2+^ concentration. The sustained increase of Ca^2+^ level feeds back to enhance cytochrome c release and amplification of apoptotic signals [29]. ER-mitochondrial Ca^2+^ signaling plays a key role in the increased apoptosis found in kidney disease. In diabetic nephropathic mice the impaired activity and expression of SERCA2 causes ER Ca^2+^ depletion, which triggers mitochondria-mediated apoptotic pathways [28]. Downregulation of mitochondrial Ca^2+^ channel polycystin 2 enhances the expression of ER-mitochondrial tethering protein mitofusin 2, which increases ER-mediated mitochondrial Ca^2+^ transfer and apoptosis and thereby contributes to ADPKD [30]. In bilateral I/R injury kidney, ER protein sigma-1 receptor (Sig-1R) dissociates from BiP and binds to ER Ca^2+^ release channel IP3R3 at ER–mitochondrial contact sites, while stabilized IP3R3 prolongs Ca^2+^ signaling to the mitochondria and enhances apoptosis [31]. These results suggest that there is a complex regulating Ca^2+^ signaling between the ER and mitochondria which is associated with apoptosis and plays an important role in kidney disease.

### 2.3. Ca^2+^ Mediates Eryptosis in Kidney Injury

Eryptosis, also known as suicidal erythrocyte death, is characterized by cell shrinkage and phosphatidylserine (PS) exposure at the erythrocyte surface. Eryptotic cells are phagocytosed and thus rapidly cleared from circulating blood. Eryptosis is the key event in eliciting renal anemia associated with CKD and anemia in end stage renal disease [32]. The mycotoxin ochratoxin A, an agent responsible for endemic Balkan nephropathy, is known to trigger anemia with suicidal cell death or eryptosis of erythrocytes by Ca^2+^-entry and increased cytosolic Ca^2+^ levels [33]. In addition, the uremic toxin indoxyl sulfate also induces suicidal erythrocyte death by stimulating extracellular Ca^2+^ entry, which may contribute to anemia in end stage renal disease [34,35]. Vanadate VO_4_^3−^ intoxication in the kidney also induces eryptosis through increased cytosolic Ca^2+^ levels, which could contribute to the development of anemia in chronic renal failure [36]. A vitamin D-rich diet in mice can augment the stimulation of PS exposure and cell shrinkage without significantly modifying the Ca^2+^ concentration in freshly drawn erythrocytes, suggesting that the effect of vitamin D treatment is presumably not effective in stimulating Ca^2+^-entry [37]. These studies indicate that dramatic Ca^2+^ entry or increasing cytosolic Ca^2+^ levels could contribute to eryptosis, which consequently causes kidney disease.

### 2.4. Ca^2+^ Signaling Regulates Autophagy in Kidney Diseases

Macroautophagy/autophagy, an evolutionarily conserved process in eukaryotes, plays an important role in intracellular material recycling. In the process of autophagy, some damaged organelles and harmful proteins are wrapped by the autophagosomes with a double membrane structure, then sent into lysosomes or vacuoles for degradation and reuse [38]. Ca^2+^, as an important messenger molecule that regulates cell death, is also involved in the regulation of autophagy [39].

It has been suggested that intracellular Ca^2+^ signaling mediates autophagy in renal tubular cells. An in vivo study has shown that the key regulator of the autophagy pathway, mTOR, is involved in tubular repair after AKI [40]. In conditionally immortalized proximal tubular epithelial cells (ciPTEC) generated from an ADPKD1 patient, activation of CaSR increased intracellular Ca^2+^ release and decreased mTOR activity [41]. Increased Ca^2+^ influx in renal proximal tubular cells inhibits mTOR-dependent autophagy, thereby rendering cells more susceptible to death [42]. This indicates that the mTOR-dependent autophagy regulated by intracellular Ca^2+^ release or Ca^2+^ influx controls the development of kidney disease.

The canonical transient receptor potential channel 6 (TRPC6), a major Ca^2+^ influx channel in renal cells, plays an important role in such renal diseases as diabetic nephropathy, immune-mediated kidney disease, renal fibrosis, glomerular disease and CKD [43]. An in vitro study in renal proximal tubular cells showed that the cytoprotective role of autophagy was suppressed by TRPC6-mediated Ca^2+^ influx [42]. The same study showed that TRPC6 knockout promoted autophagy flux and alleviated tubular apoptosis upon renal I/R, a major cause of AKI [44]. The transient receptor potential non-selective cation channel, subfamily M, member 3 (TRPM3) is another Ca^2+^ channel that conducts Ca^2+^ flux to regulating autophagy. Increased expression of TRPM3 leads to Ca^2+^ influx and stimulates autophagy through the CAMKK2/AMPK/ULK1 pathway, which promotes the growth of clear cell renal cell carcinoma (ccRCC) [45]. These results suggest that Ca^2+^ channels on the cell membrane mediate Ca^2+^ influx and suppress autophagy, which may contribute to kidney injury or diseases.

In addition, intracellular modulators of Ca^2+^ could mediate autophagy, and play important roles in kidney disease. Stromal interaction molecule 1 (STIM1) is a key regulator of ER Ca^2+^ which can activate store-operated Ca^2+^ entry (SOCE) upon sensing ER Ca^2+^ depletion. In podocytes cultured in the serum of diabetic nephrotic rats, silencing STIM1 can reverse decreased autophagy and increased epithelial–mesenchymal transition (EMT) by restoring Ca^2+^ homeostasis [46]. In podocyte cells of ADPKD, autophagy is defective and EMT is enhanced in cystic kidneys, which could be reversed by reducing ER Ca^2+^ release to silence high levels of STIM1 [47,48,49]. In epithelial kidney cells of ADPKD patients, autophagy is inhibited because of the lack of interaction between BECN1 and mutant PKD2 (encoding polycystin 2, PC2), which prolongs the increase in intracellular Ca^2+^ levels [50]. The apolipoprotein L (APOL) could function as an ion channel in intracellular membranes and be involved in regulating programmed cell death. APOL1 variants interfere with autophagy by antagonizing Ca^2+^-dependent binding of APOL3 to neuronal calcium sensor 1 (NCS-1) and interacting with PI4-kinase IIIB in podocytes, which aggravates kidney inflammation [51]. The roles of these intracellular modulators of Ca^2+^ suggests that there are also complex networks of intracellular Ca^2+^-regulated autophagy in kidney disease.

### 2.5. Lysosomal Ca^2+^ Signaling in Kidney Diseases

Lysosomal Ca^2+^ contributes to autophagy and is important for lysosomal degradation. Lysosomal degradation is mediated by a class of Ca^2+^-activated proteases, Calpains (CAPNs), on lysosomes [52]. Lack of PKD1 impairs lysosomal acidification in a CAPN protease-dependent manner, and loss-of-function mutations in PKD1 or PKD2 result in ADPKD [53]. However, in cystinotic ciPTEC lines lysosomal Ca^2+^ stores are unaffected, while some extracellular agonist-induced Ca^2+^ responses may contribute to disease pathogenesis [54]. Therefore, whether lysosomal Ca^2+^ signaling contributes to the development of kidney diseases also depends on renal cell type.

In addition, intracellular Ca^2+^ distribution between the ER, mitochondria and lysosomes may affect the function of lysosomal degradation. The mitochondria contact the ER membrane through the mitochondria-associated membrane (MAM), which provides communication between these two organelles and mediates Ca^2+^ transfer from the ER to the mitochondria. Moreover, MAM-mediated ER–mitochondria Ca^2+^ crosstalk regulates apoptosis and autophagy, which contributes to the pathogenesis of kidney disease [55]. It has been shown that Ca^2+^ redistribution from the ER to the mitochondria regulates apoptosis and autophagy and contributes to lead-induced nephrotoxicity in primary rat proximal tubular cells [56,57]. Along with the Ca^2+^ release channel IP3R and the Ca^2+^ reuptake pump SERCA, the ER delivers cytosolic Ca^2+^ to the lysosomes in “piston-like” fashion [58]. Ca^2+^ uptake by lysosomes is speculated to regulate lysosomal pH [59]. The lysosomal acidic environment is mainly regulated by lysosome V-ATPase, and has been supposed to be regulated by a Ca^2+^/H^+^ exchange, although this has not been identified in mammalian cells [60,61]. In hepatic cell HepG2, Cd induced Ca^2+^ release from the ER store to the cytosol, which caused disruption of the lysosomal acidic environment, resulting in degradation. However, the Cd-disrupted lysosomal pH was restored by pretreatment with the IP3R inhibitor 2-APB and the SERCA activator CDN1163 [62,63]. This suggests that Ca^2+^ mediates interaction between the ER and mitochondria and lysosomes, and modulates lysosomal function.

It has also been suggested that Ca^2+^ efflux from the ER regulates lysosomal Ca^2+^ levels. The ER Ca^2+^ channel-like protein transmembrane BAX inhibitor motif containing 6 (TMBIM6) not only mediates ER stress response and apoptosis, but also regulates the local release of Ca^2+^ through the lysosomal transient receptor potential mucolipin 1 (TRPML1) channel, triggering autophagy induction in the kidney in starved mice [64]. In addition, lysosomal TRPML1 also regulates mitochondria–lysosome contact and promotes Ca^2+^ transfer from lysosome to mitochondria to modulate mitochondrial homeostasis [65]. Moreover, TRPML1-mediated lysosomal Ca^2+^ release regulates transcription factor EB (TFEB), which transcriptionally regulates the expression of TRPML1 as well as expression of other autophagic and lysosomal genes [66,67]. In primary proximal tubular cells, Cd induces lysosomal dysfunction via TFEB-dependent lysosomal degradation, which leads to persistent activation of Nrf2 and causes kidney injury [68,69,70]. In addition, in the study of a knockout mouse strain (Asah1fl/fl/PodoCre) with a podocyte-specific deletion of the α subunit of acid ceramidase, lysosomal Ca^2+^ release through TRPML1 channel was inhibited in the podocytes, which may show a contribution to the development of podocytopathy and associated nephrotic syndrome [71]. These results suggest that lysosomal Ca^2+^ is regulated in the intracellular Ca^2+^ store system, which may play important roles in the progression of kidney disease.

## 3. Ca^2+^ Signaling Links Cell Death and Autophagy in Kidney Cells

The relation of cell death and autophagy is complex and occasionally contradictory, but it is critical to cell fate. Intriguingly, Ca^2+^ signaling acts as a bridge linking these two types of cellular activities [72,73,74,75,76]. Ca^2+^ promote cell proliferation and survival through release of IP3R by the ER; Ca^2+^ is subsequently transferred to mitochondria to activate mitochondrial metabolism. Mitochondrial Ca^2+^ homeostasis dysfunction results in mitochondrial degradation by autophagy via activation of AMPK. Mitochondrial Ca^2+^ overload causes production of reactive oxygen species (ROS) and release of cytochrome c, which eventually leads to cell apoptosis [39,77,78]. Therefore, Ca^2+^ signaling and Ca^2+^ subcellular homeostasis may determine the balance between cell survival, apoptosis and autophagy.

### 3.1. Induced Autophagy Promotes Cell Death

In some cases, induced autophagy promotes cell death. In the renal fibrosis model of unilateral ureteral obstruction (UUO) in mice, persistent autophagy in kidney proximal tubules was observed. Pharmacological inhibition of autophagy and kidney proximal tubule-specific knockout of autophagy-related 7 (PT-ATG7 KO) suppressed tubular atrophy, apoptosis, nephron loss, and interstitial macrophage infiltration in these mice [79]. This suggests that persistent induction of autophagy in kidney proximal tubules promotes renal interstitial fibrosis during UUO. In addition, influx of extracellular Ca^2+^ triggered by Trichokonin VI, an antimicrobial peptide, induces autophagy and apoptosis in hepatocellular carcinoma cells. Moreover, siRNA knockdown of autophagy related gene (ATG5) reduces cell apoptosis [73]. This shows that Cd induces the mitochondrial-derived autophagic cell death of hepatocytes in a dose-dependent manner. By suppressing Cd-induced autophagic cell death, melatonin has a hepatoprotective effect in Cd-exposed mice [80]. In mouse spleen and human B cells, Cd induces vacuole membrane protein 1 (VMP1)-mediated autophagy via elevation of intracellular Ca^2+^, which contributes to apoptosis [81]. In RAW264.7 mouse monocytes, Cd induced autophagy and ER-mediated apoptosis; however, pharmacological and genetic inhibition of autophagy suppressed Cd-induced apoptosis. Moreover, treatment with Ca^2+^ chelators completely restored cell viability and inhibited Cd-induced apoptosis and autophagy [82]. In porcine kidney cell LLC-PK1, the autophagy mediator calpain induced necrosis before apoptosis by increasing intracellular Ca^2+^ levels in high-glucose conditions [83]. In addition, Ca^2+^ also plays important roles in ferroptosis [84], a type of autophagy-dependent cell death [85] which has recently been shown to have implications in diverse kidney diseases [86]. These studies suggest that intracellular Ca^2+^ signaling-mediated autophagy may promote cell death and contribute to kidney disease.

### 3.2. Inhibition of Autophagy Promotes against Cell Death

In some cases, induced autophagy has protective roles against cell death. It has been shown that high glucose promotes autophagy flux in podocyte cultures and induces LC3B-II expression in podocytes in diabetic mice. Specifically, deletion of ATG5 in podocytes resulted in accelerated diabetes-induced podocytopathy with a leaky glomerular filtration barrier and glomerulosclerosis. Furthermore, the endothelial-specific deletion of ATG5 also resulted in capillary rarefaction and accelerated diabetic nephropathy. Thus, endothelial cell and podocyte autophagy synergistically protect from diabetes-induced glomerulosclerosis [87]. In mouse mesangial cells (MES-13), Cd induced autophagy and apoptosis by inducing ER Ca^2+^ release through IP3R [88]. In addition, Cd induced the expression of LC3B-II but reduced the expression of sequestosome-1 (p62) in rat mesangial cells. When autophagy is disrupted either by gene knockout or RNA silencing, cell viability is decreased and increased pro-caspase-3 cleavage indicates the initiation of apoptotic cell death [89]. These results suggest that induced autophagy may protect against nephrotoxicity. 

However, initial autophagic protection will switch to disruption of autophagic flux, resulting in cell death in renal cells [61]. In other words, inhibition of autophagy contributes to cell death. The autophagy inhibitor 3-methyladenine exacerbates Cd-caused germ cell apoptosis, which is relieved by the autophagy inducer rapamycin. More importantly, loss of ATG5 in Sertoli cells aggravates Cd-triggered germ cell apoptosis. This suggests that autophagy in Sertoli cells protects against Cd-induced germ cell apoptosis in mouse testes [90]. In human placental trophoblasts and mouse placenta, it has also been shown that activation of autophagy inhibits Cd-triggered apoptosis [91]. As an important excretory organ, the kidney is the main accumulation target of toxins such as heavy metals [92,93]. Previous studies have shown that Cd induces kidney injury and apoptosis via long-term inhibition of autophagy flux [94]. In vitro studies also show that inhibition of autophagy flux can aggravate cell apoptosis; Ca^2+^ signaling may link these two cell activities. In mouse renal tubular cells, Cd-inhibited autophagy flux aggravated apoptosis by inducing elevation of Ca^2+^ level [95]. In primary rat proximal tubular cells, Cd and lead (Pb)-inhibited autophagic degradation aggravated apoptotic death [96], which could be due to the redistribution of subcellular Ca^2+^ between the ER, cytosol and mitochondria [97,98]. Activation of CaSR can promote cell proliferation, and protects against Cd-induced renal tubular cell apoptosis through competing PLC-IP3-Ca^2+^ signaling [95]. Restoring the Ca^2+^-mediated autophagy process can protect against heavy metal-induced renal cell cytotoxicity and kidney injury [94,99,100]. These results suggest that elevation of cytosolic Ca^2+^ level-mediated inhibition of autophagy may aggravate apoptosis and contribute to kidney injury.

## 4. Targeted Ca^2+^ Signaling for Therapy of Kidney Diseases

As described above, the Ca^2+^ microdomains regulating apoptosis, necrosis and autophagy contribute to the development of kidney disease. Under these conditions, Ca^2+^ signaling determines the fate of kidney cells and the progression of disease. In view of this, therapeutic strategies have to consider whether to target a specific microdomain of Ca^2+^ signaling in kidney disease.

**Figure 1:**
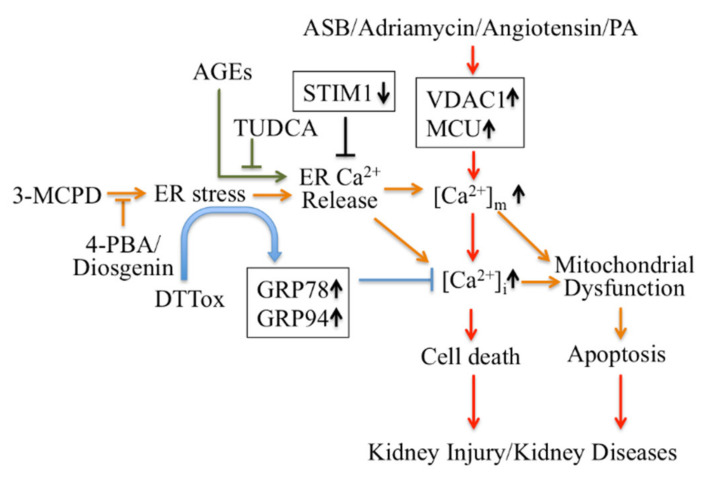
Targeting Ca^2+^ signaling regulating cell death for therapy of kidney diseases. 4-PBA: 4-phenylbutyric acid; AGEs: advanced glycation end products; ASB: andrographolide sodium bisulfite; DTTox: trans-4,5-dihydroxy-1,2-dithiane; GRP: glucose-regulated protein; PA: Palmitic acid PCV2: Porcine Circovirus Type 2; TUDCA: tauroursodeoxycholic acid; VDAC1: voltage-dependent anion channel.

Firstly, ER Ca^2+^ signaling-mediated apoptotic pathways in kidney disease could be considered as potential therapeutic targets (Figure 1). Regulating ER stress in kidney cells may provide a therapeutic target in acute kidney injury triggered by renal ischemia reperfusion and cisplatin nephrotoxicity [101]. Pretreatment with trans-4,5-dihydroxy-1,2-dithiane (DTTox) enhances expression of the ER stress markers glucose-regulated protein 78 (GRP78) and GRP94 and protects against increased cellular Ca^2+^ levels and cell death in LLC-PK1 renal epithelial cells [102], as well as protecting the kidneys from nephrotoxic injury in vivo [103]. Treatment with tauroursodeoxycholic acid (TUDCA) prevents advanced glycation end product (AGE)-induced apoptosis of mouse podocytes in diabetic nephropathy by blocking an ER Ca^2+^ release-mediated apoptotic pathway [104]. By knocking down STIM1 levels, it can reduce Ca^2+^ release and restore intracellular Ca^2+^ homeostasis, which decreases PC2 protein levels in PC1-null proximal tubule cells and inhibits cyst growth in ADPKD [49].

Secondly, modulators targeting mitochondrial Ca^2+^ mediate apoptotic pathways and may also be treatments for kidney disease therapy (Figure 1). Inhibition of ER stress by 4-phenylbutyric acid (4-PBA) and Diosgenin mitigates ER-associated mitochondrial apoptosis by maintaining Ca^2+^ homeostasis and mitochondrial dynamics, which ameliorates 3-MCPD-induced kidney injury [105,106]. In addition, 4-PBA also decreases Porcine Circovirus Type 2 (PCV2) infection-induced apoptosis by decreasing the cytosolic and mitochondrial Ca^2+^ load in porcine kidney PK-15 cells [107]. Mitochondrial outer membrane-located voltage-dependent anion channel (VDAC1) acts as gatekeeper for Ca^2+^ distribution between the mitochondria, cytosol and ER. Proteomic identification showed VDAC1 to be one the mitochondrial targets involved in andrographolide sodium bisulfite (ASB)-induced nephrotoxicity in a rat model [108]. Palmitic acid (PA) induced apoptosis through disruption of calcium homeostasis in mice podocytes [109,110]. In addition, Palmitic acid induced a continual increase in autophagy, ER stress, and apoptosis in primary cultured proximal tubular cells, and markedly upregulated VDAC1, which is associated with mitochondrial damage in HK-2 cells and may contribute to tubular injuries in obesity-related kidney disease [111]. In Adriamycin- or angiotensin II-treated rats, expression of VDAC1 and mitochondrial calcium uniporter (MCU) were upregulated; these mediate podocyte apoptosis by facilitating mitochondrial Ca^2+^ overload. However, MCU inhibitors can protect podocytes from apoptosis and proteinuria induced by Adriamycin or angiotensin II [112]. This suggests that regulating mitochondrial Ca^2+^ may also be a potential target for some stress-induced nephropathies. 

**Figure 2:**
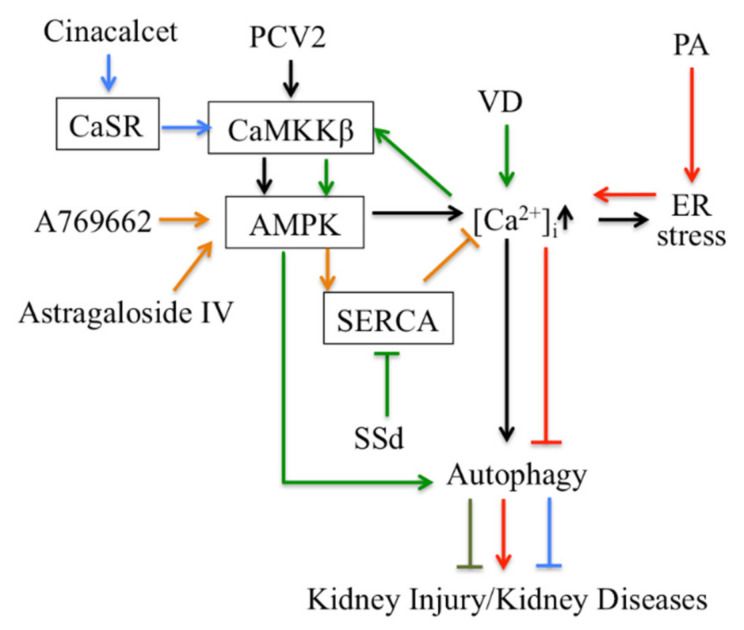
Targeting autophagy regulated by Ca^2+^ signaling for therapy in kidney disease. CaSR: Calcium sensing receptor; PA: Palmitic acid; PCV2: Porcine Circovirus Type 2; SSd: Saikosaponin-d; VD: vitamin D.

Thirdly, Ca^2+^ signaling-mediated autophagy provides new therapeutic ways to treat kidney disease (Figure 2). PCV2 induces autophagy via the CaMKKβ-AMPK pathway in pig kidney PK-15 cells by increasing cytosolic Ca^2+^ [113]. Activation of vitamin D receptors can restore defective autophagy through the Ca^2+^-CAMKKβ-AMPK pathway in renal tubular epithelial cells in streptozotocin-induced diabetic mice [114]. Ca^2+^ signaling is involved in AMPK-mediated autophagy, and plays a role in coordinating cellular survival and kidney function. A selective activator of AMPK (A769662) reduced intracellular Ca^2+^ by activation of SERCA in vascular smooth muscle, which produces vasodilation in human intrarenal arteries [115]. This suggests that activation of AMPK and SERCA might be therapeutic targets in kidney diseases. Podocyte apoptosis induced by diabetes or high glucose and progression of diabetic nephropathy are prevented by astragaloside IV, which attenuates SERCA2-dependent ER stress and induces AMPKα-promoted autophagy [116]. Saikosaponin-d (SSd), a SERCA inhibitor, suppresses excess ER Ca^2+^ reuptake and cell proliferation in ADPKD cells by inducing autophagy through the CaMKKβ-AMPK-mTOR signaling pathway, which indicates that SSd might be a potential treatment for ADPKD and that SERCA might be a novel target for ADPKD therapy [117]. It has been reported that AMPK activation restored the defective autophagy in high glucose-induced HK-2 cells [114]. Activators of CaSR such as cinacalcet have renoprotective effects in high glucose-treated human glomerular endothelial cells, murine podocytes and diabetic mice. Cinacalcet decreases oxidative stress and apoptosis and increases autophagy by increasing intracellular Ca^2+^ level through activation of the CaMKKβ-LKB1-AMPK pathway in glomerular endothelial cells and podocytes in the kidney [23]. Taken together, Ca^2+^ signaling-mediated autophagy might be a potential target in therapy for metabolic disease-associated kidney diseases.

## 5. Conclusions

Taken together, the kidney plays a key role in regulating Ca^2+^ homeostasis in the body; thus, disruption of Ca^2+^ homeostasis will cause a series of kidney diseases. Regulation of Ca^2+^ signaling in kidney cells decides cell fate in a way that is closely associated with kidney disease. Ca^2+^ microdomains including the ER, mitochondria and lysosomes regulate the various modes of cell death, such as necrosis, apoptosis and eryptosis, which when disrupted contributes to the overall development of kidney disease. Moreover, interplay between the Ca^2+^ microdomains also mediates the crosstalk between these forms of cell death and autophagy. Based on the roles of the Ca^2+^ microdomains in regulating cell fate in kidney cells, targeting these Ca^2+^ signaling may lead to novel therapeutic strategies against kidney diseases.

## Data Availability

Not applicable.
